# *Lactobacillus plantarum* MYS6 Ameliorates Fumonisin B1-Induced Hepatorenal Damage in Broilers

**DOI:** 10.3389/fmicb.2017.02317

**Published:** 2017-11-22

**Authors:** B. V. Deepthi, Rakesh Somashekaraiah, K. Poornachandra Rao, N. Deepa, N. K. Dharanesha, K. S. Girish, M. Y. Sreenivasa

**Affiliations:** ^1^Department of Studies in Microbiology, University of Mysore, Mysuru, India; ^2^Animal Disease Diagnostic Laboratory and Information Centre, Institute of Animal Health and Veterinary Biologicals, Karnataka Veterinary, Animal and Fisheries Sciences University (KVAFSU), Mysuru, India; ^3^Department of Studies and Research in Biochemistry, Tumkur Universty, Tumkur, India

**Keywords:** Fumonisin B1, *Lactobacillus plantarum*, poultry, oxidative stress, hepatotoxicity, nephrotoxicity

## Abstract

Fumonisin B1 (FB1), a mycotoxin produced by *Fusarium* species is a predominant Group 2B carcinogen occurring in maize and maize-based poultry feeds. It is shown to be nephrotoxic, hepatotoxic, neurotoxic, and immunosuppressing in animals. In this study, we report the ameliorating effects of a probiotic strain, *Lactobacillus plantarum* MYS6 on FB1-induced toxicity and oxidative damage in broilers. A 6-week dietary experiment consisting of 48 broilers was performed in six treatment groups. Probiotic treatment (10^9^ cells/mL) involved pre-colonization of broilers with *L. plantarum* MYS6 while co-administration treatment involved supplementation of probiotic and FB1-contaminated diet (200 mg/Kg feed) simultaneously. At the end of the treatment period, growth performance, hematology, serum biochemistry, and markers of oxidative stress in serum and tissue homogenates were evaluated in all the broilers. The histopathological changes in hepatic and renal tissues were further studied. The results demonstrated that administration of *L. plantarum* MYS6 efficiently improved the feed intake, body weight and feed conversion ratio in broilers. It mitigated the altered levels of hematological indices such as complete blood count, hemoglobin, and hematocrit. Serum parameters such as serum glutamic oxaloacetic transaminase, serum glutamic pyruvic transaminase, creatinine, cholesterol, triglycerides, and albumin were significantly restored after administering the probiotic in FB1-intoxicated broilers. Additionally, *L. plantarum* MYS6 alleviated the levels of oxidative stress markers in serum and tissue homogenate of liver. The histopathological data of liver and kidney further substantiated the overall protection offered by *L. plantarum* MYS6 against FB1-induced cellular toxicity and organ damage in broilers. Our results indicated that co-administration of probiotic along with the toxin had better effect in detoxification compared to its pre-colonization in broilers. Collectively, our study signifies the protective role of *L. plantarum* MYS6 in ameliorating the FB1-induced toxicity in the vital organs and subsequent oxidative stress in broilers. The probiotic *L. plantarum* MYS6 can further be formulated into a functional feed owing to its anti-fumonisin attributes and role in mitigating FB1-induced hepatorenal damage.

## Introduction

Fumonisin B1 (FB1), a potentially hazardous mycotoxin, produced mainly by *Fusarium verticillioides* and *F. proliferatum*, is a common contaminant of maize-based poultry feeds contributing to the unpalatability of feed and reduction in nutrient quality. It affects the alimentary value and organoleptic characteristics of feeds resulting in decreased feed intake and animal performance. FB1 entails the risk of mycotoxicoses and other major diseases in farm animals such as poultry (nephrotoxic and immunosuppressing effects), horses (leukoencephalomalacia), swine (pulmonary edema), cattle etc (Fandohan et al., 2005). High exposure to FB1 is hepatotoxic, and also causes lesions in gastrointestinal tract (Escriva et al., 2015). FB1 has been epidemiologically associated with cancer in humans and classified as Group 2B carcinogen by the International Agency for Research on Cancer (International Agency for Research on Cancer [IARC], 2002). The molecular aspects of FB1-induced toxicity are poorly understood, however, the downstream toxic cellular mechanisms of FB1 have been deduced to be complex involving many molecular sites. Studies have ascribed FB1-induced toxicity to the structural similarity between fumonisins and the sphingoid bases (sphinganine and sphingosine) of sphingolipid layer in cell membrane. FB1 inhibits the synthesis of ceramide by specifically binding to the sphinganine and sphingosine *N*-acetyltransferase enzymes thus deregulating the sphingolipid complex formation (Merrill et al., 2001; Riley et al., 2001; Enongene et al., 2002). This leads to the intracellular accumulation of sphingoid bases which further mediate cytotoxicity, apoptosis, cell proliferation, carcinogenicity, DNA damage (Riley et al., 2001; Voss et al., 2007), and oxidative stress (Poersch et al., 2014; Abdellatef and Khalil, 2016). In addition, Domijan and Abramov (2011) demonstrated that FB1 inhibited the complex 1 of mitochondrial electron transport chain in the cell cultures of rat primary astrocytes and human neuroblastoma (SH-SY5Y). This led to a reduction in the rate of mitochondrial and cellular respiration, depolarization of mitochondrial membrane, over production of reactive oxygen species (ROS) in mitochondria, and deregulation of calcium signaling.

Safe elimination of fumonisins from feeds and/or poultry is of paramount importance as the poultry sector suffers great economic losses due to fumonisins compared to other livestock industries. The most commonly employed detoxification method in poultry industry is the use of mycotoxin binders in feed. Nevertheless, a much promising alternative strategy would be the use of microorganisms such as lactic acid bacteria (LAB) having the potential to detoxify fumonisins. LAB constitutes an important group of probiotic organisms used as dietary supplements for humans and animals. Probiotic strains of LAB should be capable of adhering to the host intestinal epithelial cells and surviving in the gastrointestinal conditions. Beneficial aspects of probiotic LAB include increased feed conversion (Cenesiz et al., 2008), enhancement of host immune system, competitive exclusion of pathogens (Chiu et al., 2007), binding of toxic compounds (Niderkorn et al., 2007), synthesis of pathogen inhibitory metabolites (Todorov et al., 2008; Gerez et al., 2013), and mitigation of stress conditions (Poersch et al., 2014; Abdellatef and Khalil, 2016).

Poultry farming is one of the major agricultural sectors in India contributing greatly to the economy. The majority of studies on mycotoxins from India are aflatoxin-oriented, with less attention being paid to fumonisin B1 contamination in poultry feeds and their toxicity in broilers. A few studies from India demonstrated FB1 toxicity in Japanese quail (Asrani et al., 2006; Sharma et al., 2008). In the present study, we evaluated the *in vivo* potential of a probiotic LAB strain (previously characterized in our laboratory) in ameliorating the FB1-induced toxicity and oxidative stress in broilers.

## Materials and Methods

### Chemicals

All the chemicals used in this study were of the highest purity grade available commercially. Dihydrodichlorofluorescein diacetate (DCFDA) and 4-(2-hydroxyethyl) 1-piperazine ethane sulfonic acid (HEPES) were obtained from Sigma (St. Louis, United States). 2, 4-dinitrophenylhydrazine (DNPH), homovanillic acid (HVA), thiobarbituric acid (TBA), and all other chemicals and solvents were purchased from Sisco Research Laboratories (Mumbai, India). Serum glutamic oxaloacetic transaminase (SGOT), serum glutamic pyruvic transaminase (SGPT), albumin, triglycerides, creatinine, and cholesterol commercial kits were purchased from Swemed Diagnostics (Bengaluru, India).

### Diet Preparation and Probiotic Strain

FB1 test diets were prepared using commercially available broiler feed (basal diet; Shresta feeds, Bengaluru). The basal diet composition is given in **Table 1**. Culture jars containing 1 kg of broiler feed (a_w_ = 1, autoclaved at 121°C for 20 min) were inoculated with 20 mL of toxigenic *Fusarium verticillioides* MTCC 1848 (10^6^ spores/mL). The jars were shaken thoroughly to ensure complete dispersal of the fungal spores and incubated in dark for 45 days at 28°C ± 2°C. After incubation, jars were dried in hot air oven at 60°C for 8–10 h. The dried feed mixture was ground in a blender to a fine meal. The finely ground feed mixture (0.4 g) was taken in a sterile amber vial and suspended in 2.0 mL acetonitrile:water (1:1) and allowed for equilibration overnight in a gel rocker at 28°C ± 2°C. The extracts were syringe filtered using 0.45 μm nylon membrane filters and subjected to liquid chromatography/mass spectrometry (LC/MS) (Waters Acquity/ Synapt G2, United States). Chromatographic separation was achieved on a C18 column maintained at 50°C. Mobile phase A was 0.3% formic acid in water (v/v) and acetonitrile being mobile phase B. The mass spectrometer was operated in the positive electronspray ionization mode (ESI+). The limit of detection (LOD) for FB1 was 10 ng/mL and retention time was found to be 1.77 min. (Deepthi et al., 2016). The cultured broiler feed was mixed with the basal feed to obtain FB1 treatment equivalent to the ingestion of diet containing toxin concentration of 200 mg/kg feed.

**Table 1:** Feed composition of the basal diet during pre-starter, starter, and finisher adopted in the experiment.

Ingredients	Pre-starter (%)	Starter (%)	Finisher (%)
Maize	55.35	59.08	63.44
Soya meal	35	32.5	27.5
Rice polish	4.8	3	3
Rice bran oil	1	2	3
Limestone powder	1.3	1.14	1.1
Digestible crude protein	0.8	0.64	0.4
Salt	0.42	0.37	0.28
Amino acids	0.51	0.48	0.45
Other supplements	0.82	0.79	0.84


The bacterial strain, *Lactobacillus plantarum* MYS6 (LpMYS6) which was previously characterized in our laboratory with respect to its probiotic and antifungal attributes (Deepthi et al., 2016) was used in this study. The bacterium was cultured in de Man Rogosa Sharpe (MRS) broth anaerobically at 37°C for 48 h. Cells were harvested by centrifugation at 8000 rpm for 10 min, washed thrice and re-suspended in phosphate buffered saline (PBS, 100 mM, pH 7.4) to a final concentration of 10^9^ cells/mL.

### Experimental Design and Treatment

The poultry trial was approved (UOM/IAEC/02/2013) by the Animal Ethical Committee, Department of Zoology, University of Mysore, Mysuru.

One-day old, 48 Cobb variety broiler chicks (male 27, female-21) were used in this study. The 1-day old chicks weighed in a range from 42 to 46 g and were reared in an environmentally controlled room for 42 days. Chicks were randomly distributed into six treatment groups with each treatment having eight chicks. The experimental design involving six treatment groups is described in **Table 2**. The basal diet types included pre-starter feed (0–7 days), starter feed (8–21 days), and finisher feed (22–42 days). Initial 1-week of acclimatization period at high temperature (90°C) was provided to one-day old chicks followed by vaccination on day 7. The experiment was performed in two ways: (a) pre-colonization study which started with the pre-incubation of LpMYS6 from day 8 followed by supplementation of FB1-contaminated feed mixture from day 12 and (b) challenge study involved the co-administration of chicks with LpMYS6 and FB1-contaminated feed simultaneously from day 12. One mL (10^9^ cells/mL) of LpMYS6 preparation was administered daily by oral gavage. The positive control consisted of a commercially available multi-spectrum mycotoxin binder (Varishta, Bengaluru, India) that was administered to the chicks from day 12. The toxin binder (TOXB) was a combination of *Picrorhiza kurroa*, activated charcoal, hydrated sodium calcium aluminosilicate (HSCAS), mannan oligosaccharide (MOS), buffered organic acids, and antioxidants. The control (C) group was also a negative control where the chicks were provided with basal diet and PBS. All the chicks were vaccinated for Newcastle disease and Infectious Bursal Disease (IBD) on day 7 and day 14, respectively. Antibiotics and liver stimulants were not supplemented in the basal diet and the basal feed was free from aflatoxins and other major mycotoxins. All the treatment groups were provided with water and basal diet *ad libitum*.

**Table 2:** Treatment groups of *in vivo* study.

Experimental groups	Treatment
Control (C)	Basal diet + PBS control
Positive Control (TOXB)	200 mg FB1/kg feed + 1.0 g/kg feed toxin binder
Group TOX	200 mg FB1/kg feed
**Pre-colonization study**	
Group LP	10^9^ cells/mL *Lactobacillus plantarum* MYS6
Group LP+TOX	200 mg FB1/kg feed + 10^9^ cells/mL *L. plantarum* MYS6
**Challenge study**	
Group TOX_LP	200 mg FB1/kg feed + 10^9^ cells/mL *L. plantarum* MYS6


### Broiler Performance and Sampling

To evaluate the influence of FB1 and LpMYS6 on broiler performance, body weight of each bird was measured at weekly interval and feed consumption of every pen was monitored throughout the experimental period. The feed conversion ratio (FCR) was calculated for each treatment on the basis of unit feed consumption to unit body weight gain. Treatment groups were observed daily to record the morbidity/mortality. On the conclusion of experiment (day 42), broilers were submitted to pre-slaughter fasting for 18 h. Five birds from each treatment group were randomly selected, weighed, euthanized by cervical dislocation and necropsied for the excision of liver and kidney. Prior to sacrifice, blood samples were collected from the jugular vein in two sets. One set for hematological study was collected in the sterile vials containing the anticoagulant citrate dextrose (ACD) maintained in ice bath. The other set of blood samples was collected in dry sterile tubes, centrifuged at 5000 rpm for 10 min and the serum was separated and stored at -20°C until further use. The excised liver and kidney tissues were blotted free of blood, rinsed with PBS, weighed and stored at -20°C for histopathological studies. The excised liver tissues were homogenized (10% w/v) in ice-cold potassium phosphate buffer (100 mM, pH 7.4) and centrifuged at 5000 rpm for 15 min at 4°C. The resulting supernatant (total liver homogenate) was stored at -20°C for the analysis of different parameters to evaluate oxidative stress.

### Determination of Hematological Parameters

Citrated blood samples of five birds from each treatment group were checked for hematological indices using a blood cell counter (ERMA PCE210, Tokyo, Japan). The following parameters were examined: hemoglobin (HGB), red blood corpuscles (RBC), white blood corpuscles (WBC), platelets (PLT), and hematocrit (HCT).

### Determination of Serum Biochemical Parameters

Serum samples were screened for routine biochemical parameters such as albumin, triglycerides, creatinine, and cholesterol. Liver function was assessed by the activities of serum glutamic oxaloacetic transaminase (SGOT) and serum glutamic pyruvic transaminase (SGPT). All the above parameters were estimated according to the instructions of assay kits (Swemed Diagnostics, Bengaluru, India) using a versatile biochemistry analyzer ARTOS-SBPL/418 (Swemed Diagnostics, Bengaluru, India).

### Analysis of Oxidative Stress Markers

#### Estimation of Reactive Oxygen Species (ROS)

The levels of endogenously generated ROS were measured in liver homogenate and serum as per the protocol described by Driver et al. (2000). In this assay, an aliquot of liver homogenate (20 μL, 0.5 mg protein) was dispensed into a 96 well microtitre plate containing 170 μL of Locke’s buffer (154 mM NaCl, 5.6 mM KCl, 3.6 mM NaHCO_3_, 5 mM HEPES, 10 mM glucose, 2 mM CaCl_2_, pH 7.4). To this mixture, 10 μL of DCFDA (10 μM) was added and incubated at room temperature for 30 min. After incubation, fluorescence was measured using a multimode plate reader (Thermo Scientific, United States) with excitation and emission at 480 nm and 530 nm, respectively. To measure the ROS levels in serum, the same assay was followed but the liver homogenate was replaced with 10 μL of serum. Background fluorescence was corrected by the inclusion of parallel blanks. A dichlorofluorescein standard curve was used to quantify ROS levels and the data were expressed as pmol DCF formed per mg protein.

### Estimation of Hydrogen Peroxide Level

Another well-known oxidative stress marker, hydrogen peroxide (H_2_O_2_) was quantified in liver homogenate and serum as per the method followed by Botsoglou et al. (2010) with minor modifications. Here, 20 μL (0.5 mg protein) of liver homogenate was dispensed into 96 well microtitre plate containing HEPES buffered saline (HBS, 145 mM NaCl, 10 mM HEPES, 10 mM glucose, 5 mM KCl, 1 mM MgSO_4_, pH 7.4) and incubated with 100 μM HVA at room temperature for 45 min. After incubation, reaction mixture was excited at 312 nm and fluorescence was measured at 420 nm. The H_2_O_2_ levels in serum samples were measured by using 10 μL serum as incubation mixture instead of liver homogenate. The H_2_O_2_ levels were expressed as nmol H_2_O_2_ per mg protein.

#### Assessment of Lipid Peroxidation (LPO)

Lipid peroxidation (LPO) status was assessed in liver homogenate and serum samples by measuring thiobarbituric acid reactive substances (TBARS) and was expressed in terms of malondialdehyde (MDA) content, as described by Ohkawa et al. (1979). In this assay, tubes containing 1.5 mL acetic acid (20% v/v, pH 3.5), 0.2 mL SDS (8% w/v), 1.5 mL TBA (0.8% w/v) were added with 200 μL of test sample (liver homogenate/serum). The reaction mixture was incubated in a boiling water bath for 45 min. After cooling to room temperature, 3 mL of butanol was added to extract adducts formed and were centrifuged at 2000 rpm for 10 min. The absorbance was measured in the supernatant at 532 nm and results were expressed in terms of malondialdehyde equivalents as nmol MDA formed per mg protein.

#### Measurement of Protein Carbonyl Content (PCC)

Protein carbonyl content (PCC) was estimated in the liver homogenate and serum samples using DNPH following the method described by Levine et al. (1990). Two hundred microliter of sample (liver homogenate/serum; 0.5–1 mg protein) was added into eppendorf tube containing 500 μL of 10 mM DNPH in 2 N HCl and incubated at room temperature for 1 h with intermittent shaking. Corresponding blank was maintained by adding only 2 N HCl to the sample. After incubation, 500 μL 20% TCA was added to the mixture to precipitate proteins followed by centrifugation at 5000 rpm for 10 min. The precipitate was washed twice with acetone and finally suspended in 1 mL of Tris buffer (20 mM, pH 7.4 containing 140 mM NaCl and 2% SDS w/v), vortexed and incubated overnight at 4°C. The absorbance of the mixture was read at 360 nm and expressed as nmol of carbonyl groups per mg protein.

### Histopathology

The formalin-fixed tissue samples of liver and kidney were processed routinely, sectioned at 4–5 μm thickness, stained with hematoxylin-eosin dye and observed and photographed using Olympus Bx41 microscope equipped with ProgRes CT3 camera (Tokyo, Japan).

### Protein Estimation

The protein concentration of liver homogenate was estimated by the protocol of Lowry et al. (1951) using bovine serum albumin (BSA) as the standard.

### Statistical Analysis

The data obtained in this study are the mean of five determinations expressed as mean ± SEM and analyzed by one-way analysis of variance (ANOVA) followed by Bonferroni’s *post hoc* test for multiple comparison, with following probability ^∗^*P* < 0.05, ^∗∗^*P* < 0.01, and ^∗∗∗^*P* < 0.001 to be considered statistically significant. The graphs were drawn using GraphPad Prism version 5.03 software (GraphPad Software Inc.).

## Results

### Growth Performance

On completion of 42 days of feeding experiment, broilers treated with only FB1-contaminated diet (TOX) showed a feed intake 2.725 Kg when compared to the control group (2.948 Kg). Also, FCR was found to be 1.741 ± 0.06 when compared to the control treatment (1.965 ± 0.07). There was no significant difference in the body weight of FB1-intoxicated broilers (TOX) when compared to control. Broilers of pre-colonization treatment (LP and LP+TOX) showed a feed intake of 2.787 and 2.855 Kg, respectively, when compared to the TOXB group (2.933 Kg) and showed a FCR ranging from 1.845 ± 0.27 and 1.860 ± 0.07 when compared to that of TOXB group (1.777 ± 0.06). Our study showed no significant changes of body weight in the broilers of treatment groups. The body weight, feed consumption and FCR of each treatment are tabulated in **Table 3**. No mortality was recorded in any of the treatment groups during the experiment. Broilers fed with FB1 concentration alone, suffered from dysentery 5 days post toxin treatment (TOX) until the end of experiment. While the broilers of TOXB group and challenge study witnessed recurrent episodes of diarrhea during the experiment. It is important to note that, groups of pre-colonization study administered with LpMYS6 alone and along with toxin did not show any signs of illness throughout the experiment.

**Table 3:** Effect of *L. plantarum* MYS6 and fumonisin B1-supplemented feed on growth performance of broilers after 42 days of feeding experiment.

Groups	Body weight (Kg)	Feed intake (Kg)	FCR	Mortality
C	1.509 ± 0.05	2.948	1.965 ± 0.07	0
LP	1.614 ± 0.17	2.787	1.845 ± 0.27	0
TOX	1.575 ± 0.05	2.726	1.741 ± 0.06	0
LP+TOX	1.545 ± 0.06	2.855	1.860 ± 0.07	0
TOX_LP	1.918 ± 0.01^a∗^	2.902	1.507 ± 0.00^b∗^	0
TOXB	1.659 ± 0.05	2.933	1.777 ± 0.06	0


*Data are the mean ± SEM of five broiler birds per treatment group. ^∗^*P* < 0.05*; ^a^*Significant when compared to 200 mg toxin control*, ^b^*Significant when compared to TOXB group.*

Postmortem examination of organs revealed yellowish discoloration of liver in broilers fed with 200 mg FB1-contaminated diet, and also witnessed atrophy of liver (**Figure 3i**). Further, broilers of other experimental groups showed dark brown-enlarged liver when compared to normal sized liver of control group (**Figures 3a,e,m,q**). The effects of TOX on relative organs weight are presented in **Table 4**. Increase in the mean weight of liver of 3.86 ± 0.16 g/100g body weight was observed in broilers fed with 200 mg FB1-contaminated diet (TOX) compared to that of the control (2.84 ± 0.13 g/100g body weight). With respect to mean weight of kidney, substantial differences were not observed in the treatment groups in comparison to the control.

**Table 4:** Relative organ weights of control and treatment broilers after 42 days of feeding experiment.

	Relative organ weights (g/100g body weight)	
	
Groups	Liver	Kidney
C	2.84 ± 0.13	0.08 ± 0.01
LP	3.04 ± 0.35^a∗^	0.10 ± 0.00
TOX	3.86 ± 0.16^b∗∗^	0.13 ± 0.01
LP+TOX	3.69 ± 0.11	0.11 ± 0.00
TOX_LP	3.20 ± 0.09	0.11 ± 0.00
TOXB	3.61 ± 0.15	0.09 ± 0.03


*Data are the mean ± SEM of 5 broiler birds per treatment group. ^∗^*P* < 0.05*; ^a^*Significant when compared to toxin control. ^∗∗^*P* < 0.01*; ^b^*Significant when compared to control.*

### Hematological Indices

FB1 toxicity in broilers fed with 200 mg toxin damaged the hematological parameters. **Table 5** summarizes the effects of FB1 concentration and LpMYS6 on the hematological indices of broilers. The WBC and PLT count in the control group were 35420 ± 3860 cells/μL and 12000 ± 700 cells/μL, respectively. While 200 mg toxin fed broilers showed a count of WBC up to 40860 ± 850 cells/μL and recorded 9000 ± 410 cells/μL of platelet count. Further, broilers treated with LpMYS6 alone revealed a lesser count in WBC compared to all other groups including control. In case of TOXB group, challenge study and TOX of pre-colonization study, a slight non-significant increase in WBC count with that of control was observed revealing no much differences within these groups. With respect to platelet count, treatment group which received LpMYS6 alone showed no differences when compared to control. Additionally, platelet count was slightly reduced in TOXB group, challenge study and FB1 treatment group (200 mg) of pre-colonization study when compared to the control.

**Table 5:** Effect of *L. plantarum* MYS6 and fumonisin B1 supplemented feed on hematological indices of broilers after 42 days of feeding experiment.

Groups	WBC10^3^/μL	RBC10^6^/μL	Hbg/dL	HCT%	PLT10^3^/μL
C	35.42 ± 3.86	2.25 ± 0.04	9.94 ± 0.18	29.16 ± 1.96	12.0 ± 0.70
LP	33.3 ± 4.85	2.09 ± 0.39	9.1 ± 1.47	28.52 ± 6.03	12.0 ± 1.94
TOX	40.86 ± 0.85	1.52 ± 0.17^a∗^	7.16 ± 0.42^b∗^	23.6 ± 0.07	9.0 ± 0.41
LP+TOX	38.04 ± 0.97	1.94 ± 0.09	8.40 ± 0.23	24.04 ± 1.06	10.0 ± 0.31
TOX_LP	38.58 ± 1.22	2.07 ± 0.06	9.2 ± 0.08	26.12 ± 0.66	10.0 ± 0.31
TOXB	38.9 ± 1.17	1.9 ± 0.08	8.64 ± 0.30	28.46 ± 3.15	9.2 ± 0.66


*Data are the mean ± SEM of five broiler birds per treatment group. ^∗^*P* < 0.05*; ^a,b^*Significant when compared to control. WBC, white blood cells, RBC, red blood cells, Hb, hemoglobin, HCT, hematocrit, PLT, platelets.*

Broilers fed with diet containing 200 mg FB1 concentration alone revealed a reduced (^∗^*P* < 0.05) RBC count, hemoglobin concentration and per cent HCT. Broilers of pre-colonization study fed with only LpMYS6, TOXB group and challenge study showed values close to that of control. However, TOX in broilers pre-colonized with LpMYS6 reestablished the RBC count and hemoglobin concentration while the per cent HCT remained unchanged.

### Serum Biochemistry

**FIGURE 1:**
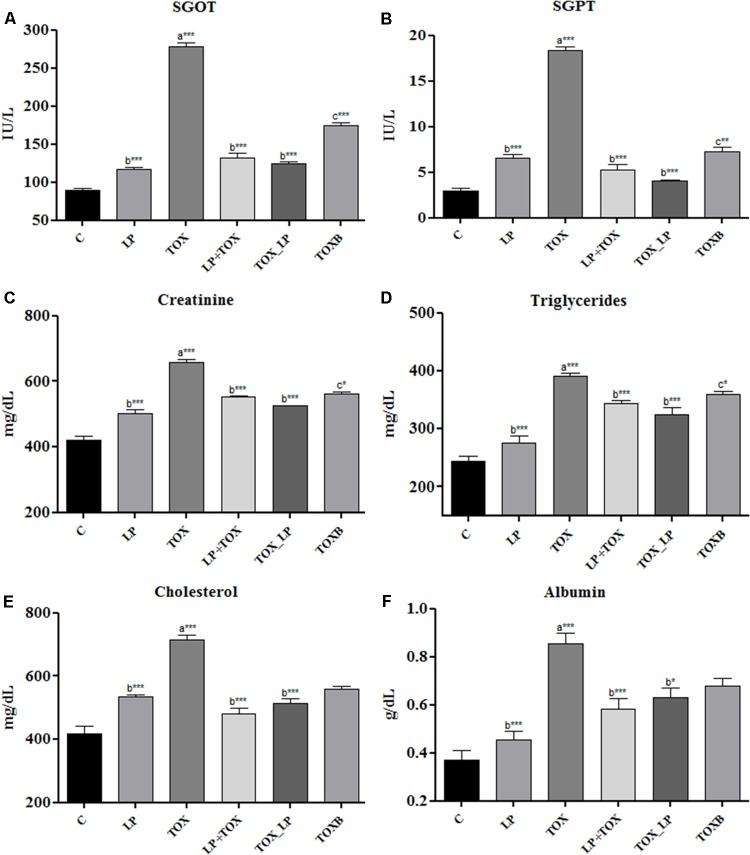
Effect of fumonisin B1, *Lactobacillus plantarum* MYS6 and toxin binder (TOXB) on biochemical parameters of control and treatment broilers. The serum levels of **(A)** SGOT, **(B)** SGPT, **(C)** creatinine, **(D)** triglycerides, **(E)** cholesterol, and **(F)** albumin. Data are the mean ± SEM of five broiler birds per treatment group and were analyzed using one way ANOWA followed by Bonferroni *post hoc* test (^∗^*P* < 0.05, ^∗∗^*P* < 0.01, ^∗∗∗^*P* < 0.001; ^a^significant when compared to control, ^b^significant when compared to 200 mg FB1 toxin control, ^c^significant when compared to challenge study).

Forty-two days post-feeding, all the biochemical parameters augmented significantly (*P* < 0.001) in the serum of broilers fed with 200 mg FB1 independently compared to control (**Figure 1**). SGOT and SGPT, were high in the TOX fed with 200 mg FB1 only (**Figures 1A,B**). Treatment with TOXB also caused a significant increase in the levels of SGOT (*P* < 0.001) and SGPT (*P* < 0.05) when compared to control (**Figures 1A,B**). In contrast, the SGOT and SGPT levels were significantly reduced in the TOX (LP+TOX) of pre-colonization study administered with LpMYS6. It is notable that, in the challenge study, LpMYS6 could significantly (*P* < 0.001) alleviate the augmented serum levels of SGOT and SGPT in comparison with TOXB group and pre-colonization study (**Figures 1A,B**).

Damage to kidney was assessed by creatinine level and is depicted in the **Figure 1C**. Here, an increase in the creatinine concentration was observed in the TOXB group and 200 mg FB1-fed groups when compared to the control. In pre-colonization and challenge study, LpMYS6 significantly (*P* < 0.001) lowered the elevated levels of creatinine, however, the reduction level had no much difference within the treatment groups (**Figure 1C**). Broilers fed with FB1 toxin alone showed a significantly (*P* < 0.001) high triglyceride, cholesterol and albumin levels in the serum compared to control (**Figures 1D–F**). TOXBs and LpMYS6 each in combination with toxin lowered slightly the high levels of triglyceride and cholesterol (**Figures 1D,E**). While in case of albumin, a significant decline was noted in broilers of pre-colonization and challenge study in comparison to TOXB group as well as broilers treated with FB1 alone (**Figure 1F**).

### Markers of Oxidative Stress

#### Generation of ROS

**FIGURE 2:**
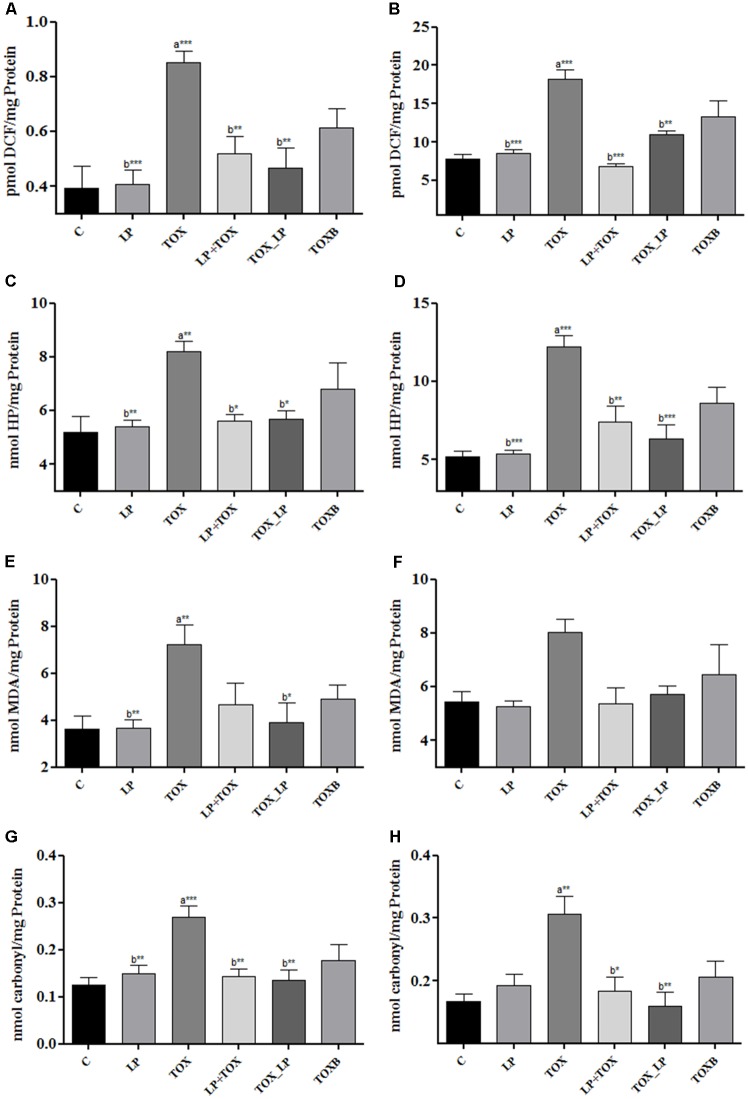
Effect of *L. plantarum* MYS6 on fumonisin B1 induced oxidative stress in the liver homogenate and serum of control and treatment broilers. In the figure, **(A,B)** generation of ROS, **(C,D)** generation of hydrogen peroxide, **(E,F)** peroxidation of lipids, and **(G,H)** oxidation of proteins. Data are the mean ± SEM of five broiler birds per treatment group and were analyzed using one way ANOWA followed by Bonferroni *post hoc* test (^∗^*P* < 0.05, ^∗∗^*P* < 0.01, ^∗∗∗^*P* < 0.001; ^a^significant when compared to control, ^b^significant when compared to 200 mg FB1 toxin control).

Our study demonstrated a significantly high generation of ROS in liver homogenate and serum of broilers fed with FB1 alone when compared to the control group. The pre-colonization of broilers with LpMYS6 remarkably reduced the levels of ROS in liver homogenate indicating a better result when compared with the TOXB group (**Figure 2A**). Also, administering broilers with LpMYS6 and 200 mg FB1 simultaneously in challenge study showed a significant (*P* < 0.01) decline of ROS in liver homogenate than other TOX groups (**Figure 2A**). Additionally, pre-colonization of broilers with LpMYS6 in 200 mg FB1 treatment (LP+TOX) resulted in a notable (*P* < 0.001) decline of ROS level in serum. This was less than the ROS of control group and all the other treatment groups (**Figure 2B**).

#### Generation of Hydrogen Peroxide

A substantial elevation of free radical H_2_O_2_ was observed in the liver homogenate (**Figure 2C**) and serum (**Figure 2D**) of TOX group (200 mg FB1) in comparison with control. An equal and effective reduction by LpMYS6 was observed in H_2_O_2_ in the liver homogenate of pre-colonization and challenge study when compared to the TOXB group (**Figure 2C**). However, the levels of reduction in H_2_O_2_ were nonsignificant between the TOXB group and challenge study. Further, LpMYS6 was more effective in lowering the elevated levels of H_2_O_2_ in the serum samples of challenge study (TOX_LP) when compared with the LP+TOX group (200 mg FB1) and TOXB group (TOXB group) at *P* < 0.001 (**Figure 2D**).

#### Lipid Peroxidation

FB1-induced LPO was estimated as an increase in the concentration of MDA in liver homogenate and serum. In the FB1-alone-treated broilers, the concentration of MDA in liver homogenate (**Figure 2E**) and serum (**Figure 2F**) were significantly high compared to the control group. LpMYS6 in pre-colonization study and its co-administration with FB1 in challenge study reduced the levels of MDA both in liver homogenate (**Figure 2E**) and serum (**Figure 2F**) compared to the TOXB-supplemented group. But the reduction level of MDA in serum was observed to be non-significant in all the groups.

#### Protein Oxidation

Carbonyl content generated as a result of protein oxidation was significantly high in the liver homogenate (**Figure 2G**) (*P* < 0.001) and serum (**Figure 2H**) (*P* < 0.01) of broilers treated with FB1 alone as compared to control group. Compared to the TOXB, LpMYS6 was more effective in reverting (*P* < 0.01) the increased levels of carbonyl content in the liver homogenate to almost normal state as that of control (**Figure 2G**). Besides, LpMYS6 fed broilers of TOX_LP group (challenge study) significantly brought down the elevated levels of PCC in serum (**Figure 2H**). Interestingly, independent administration of LpMYS6 in LP group showed a slight non-significant increase in carbonyl content of serum compared to the TOXs of pre-colonization and challenge studies (**Figure 2H**).

#### Histopathology

**FIGURE 3:**
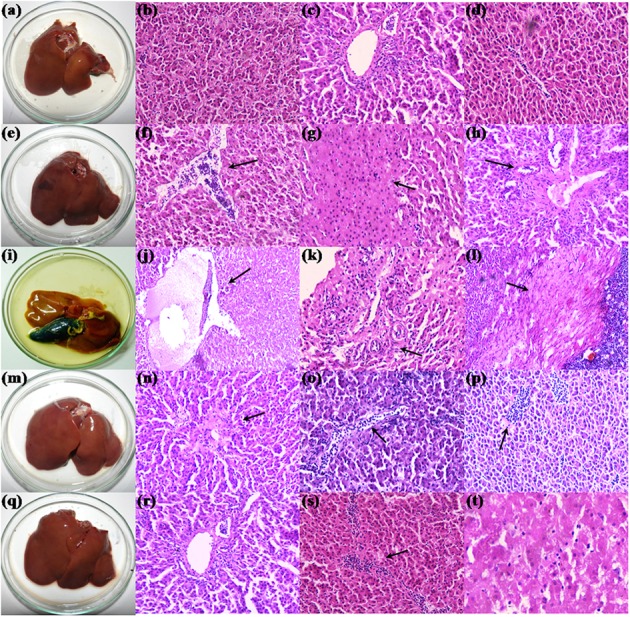
Morphology and histopathology of hematoxylin-eosin-stained photomicrographs of liver of control and treatment broilers. **(a–d)** Control, **(e–h)** TOXB + 200 mg FB1, **(i–l)** 200 mg FB1 alone, **(m–p)** *L. plantarum* MYS6 alone, and **(q–t)** LpMYS6 + 200 mg FB1. Liver sections were observed at 400× magnification.

The histopathological examination substantiated the FB1-induced toxicity in hepatic and renal parenchyma. The liver of control group showed a mild irregularity in the hepatocyte arrangement in hepatic cords (**Figures 3b,c**). Also, a mild infiltration of mononuclear cells in the periportal areas and sinusoidal space were noticed due to aging (**Figures 3c,d**). The liver of broilers treated with 200 mg FB1 alone showed severe disorganization in parenchyma compared to the control group revealing progressive stages of FB1 toxicity. Severe necrosis and edema (**Figure 3j**) of liver parenchyma was conspicuous. Biliary hyperplasia accompanied with inflammatory cells infiltration (**Figure 3k**) was evident of toxin insult. Additionally, liver tissue manifested extensive fibrosis surrounded by severe mononuclear cells infiltration suggestive of chronic inflammation (**Figure 3l**). Broilers fed with TOXBs showed a lower severity grade when compared to FB1 alone. Liver tissue of TOXB group displayed inflammatory cell infiltrates (**Figure 3f**), disorganized hepatic cords followed by hepatic necrosis (**Figure 3g**). Besides, moderate bile duct proliferation was observed in the liver of TOXB group (**Figure 3h**). The liver of broilers treated with LpMYS6 alone exhibited a mild hepatic disorganization with mild degeneration and focal to diffused infiltration of mononuclear cells (**Figures 3n–p**). However, LpMYS6 effectively reduced the degree of FB1 toxicity in broilers of pre-colonization (200 mg FB1 treatment) and challenge study. The liver displayed normal hepatocytes architecture though a mild to moderate hepatic disorganization (**Figure 3r**) was noticed. Severity of necrosis and edema was considerably attenuated (**Figure 3t**). A remarkable reduction in the focal inflammation and infiltration of mononuclear cells to the sinusoid was conspicuous (**Figure 3s**). The liver sections showed no fibrosis and biliary hyperplasia compared to the TOXB group and FB1 treatment alone.

**FIGURE 4:**
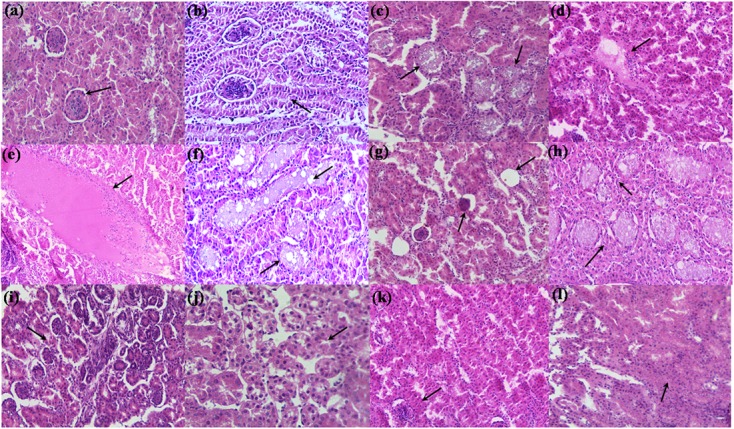
Hematoxylin-eosin stained photomicrographs of kidney of control and treatment broilers. **(a,b)** Control, **(c,d)** TOXB + 200 mg FB1, **(e–h)** 200 mg FB1 alone, **(i,j)** *L. plantarum* MYS6 alone, and **(k,l)** LpMYS6 + 200 mg FB1. Kidney sections were observed at 400× magnification.

Kidney sections of control group showed aging morphology involving normal renal glomeruli and Bowman’s capsule space (**Figure 4a**). The increased cellularity of glomeruli and mild desquamation of tubular epithelium was observed (**Figure 4b**). Kidney sections of broilers fed with TOXB showed severe disruption of tubular epithelium and accumulation of granular casts in glomeruli (**Figure 4c**). Focal inflammation, cellular infiltration and mild edema were observed in the interstitium accompanied with distal tubular damage (**Figure 4d**). While broilers fed with 200 mg FB1 alone exhibited severe desquamation of tubular epithelium followed by destruction of renal parenchyma. Sections showed damaged architecture accompanied by acute inflammation with infiltration of inflammatory cells and massive edema (**Figure 4e**). Tubular degeneration proceeded by cytoplasmic vacuolation, fragmented cytoplasm and tubular lumen filled with eosinophilic deposits were frequently encountered as the indicators of renal tubule necrosis (**Figure 4f**). Besides necrosis, cystic glomeruli (**Figure 4g**) and series of renal tubule enlargement involving granular casts and vacoulation of glomeruli (**Figure 4h**) were the other morphologic severity associated with the degeneration of tissue. In contrary, kidneys of broilers treated with LpMYS6 alone depicted a normal morphology of Bowman’s capsule in cortex and distal tubules yet age-related mild disruption of renal parenchyma was noticed (**Figures 4i,j**). Furthermore, treatment with LpMYS6 in pre-colonization and challenge study effectively restored the normal histology of kidney tissues. Renal tubule regeneration was clearly observed and included an array of histological changes such as karyomegaly and nuclear crowding along the affected renal tubules (**Figures 4k,l**). In addition, the severity grade of FB1 toxin was extensively reduced and no signs of edema, necrosis, cystic glomeruli, granular casts or any major abnormalities in the renal parenchyma were observed.

## Discussion

Fumonisin B1-induced deregulation of sphingolipid complex formation is a well-studied FB1 toxicity in animals and humans. Unlike most mycotoxins which are cyclic compounds, FB1 is a long-hydroxylated hydrocarbon chain having structural similarity to the sphingoid bases. FB1 intoxication also manifests serious consequences of oxidative stress in animals apart from the inhibition of sphinogolipid metabolism (Poersch et al., 2014). FB1 is poorly absorbed in the gastrointestinal tract unlike aflatoxins and is extensively retained as unmetabolized in the tissues such as liver and kidney. The toxin is eliminated in bile through enterohepatic recirculation and exerts its toxicity. It may also end up in the feces and as trace contaminants in urine (Shephard et al., 1994; Enongene et al., 2000). Apparently, poultry ingested with FB1 exhibit lack of appetite, low productive performance, inflamed liver, oral lesions, and immunosuppression leading to adverse health and economic status (Poersch et al., 2014). Hence protection against FB1-induced toxicity and oxidative stress in poultry becomes a prerequisite.

In our study, we observed a non-significant change in feed intake and feed conversion in the broilers treated with FB1 alone compared to control. Previous studies have reported a decrease in feed intake and feed conversion when broilers, ducks, or turkeys were ingested with feed contaminated by FB1 (Tessari et al., 2006; Benlasher et al., 2012). Further, our study showed no significant variations of body weight in broilers of treatment groups but health complications such as diarrhea and dysentery was observed in broilers fed with 200 mg FB1 per Kg feed. Earlier reports by Tran et al. (2005) and Tardieu et al. (2007) observed a reduced body weight in ducks and turkeys fed with 0–128 mg of FB1/Kg feed and 0–20 mg of FB1/Kg feed, respectively. Besides, increased relative organ weights of liver and kidney in our study marks FB1 toxicity as these organs are the sites of detoxification of many toxicants including FB1, and also the target organs for toxin effects (Voss et al., 2007). Poersch et al. (2014) also reported a significant increase in the liver weight of broilers treated with FB1 contaminated feed (100 mg/kg feed) showing its toxicity in the target organs.

With respect to hematological indices, broilers fed with 200 mg FB1-contaminated feed showed a diminished RBC count and hemoglobin (*P* < 0.05). This could be due to the toxic effects of FB1 on liver leading to haematopoietic (such as vitamins B12, folic acid, iron) suppression which eventually results in decreased synthesis of hemoglobin and erythropoietin and subsequently affect the RBC production and HCT. Our results are in accordance with the study conducted by Sobrane Filho et al. (2016), where broilers fed with diet containing a mixture of aflatoxin B1 (2 mg/kg feed) and FB1 (100 mg/kg feed) exhibited low values of erythrocyte, hemoglobin, and hematocrit compared to control while no difference was observed in the thrombocyte count. Another study by Broomhead et al. (2002) showed that the above parameters of hematology remain unaffected in broilers when treated with 25 or 50 mg/kg FB1 in diet. FB1 fed broilers in the present study showed a non-significant rise in WBC count probably due to an inflammatory response to the toxin. But Sobrane Filho et al. (2016) reported a decrease in WBC count in the broilers fed with aflatoxin B1 (2 mg/kg feed) and FB1 (100 mg/kg feed) contaminated diet. The inconsistency in the hematology results is tempting us to speculate that hematology would not be a sensitive indicator of FB1 toxicity in broilers.

Further, increased SGOT and SGPT might be the signs of damage of hepatocytes and these could be sensitive indicators of acute hepatic necrosis. In agreement with our results, Khalil et al. (2015) reported a significant increase in serum liver function markers such as alanine aminotransferase (ALT), aspartate aminotransferase (AST), and alkaline phosphatase (ALP) in Sprague–Dawley rats intoxicated with 100 and 200 mg FB1. The increased levels of cholesterol and triglyceride in our study are probable indicators of stress created in broilers due to FB1 feeding affecting lipid metabolism. Also, this could be linked to decreased feed intake and disruption of sphingolipid metabolism because of the interrelationships of these pathways. Meanwhile, a rise in the albumin in FB1-challenged broilers may be due to the damage of hepatocytes and impairment of protein synthesis. A study conducted by Cheng et al. (2006) in broilers fed with 5 and/or 15 mg FB1 showed a significant increase in serum AST, albumin, and cholesterol coupled with poor immunocompetence. Besides, increased levels of creatinine in the present study could be the result of protein catabolism or kidney affliction. A previous study by Khalil et al. (2015) reported a gradual increase in renal products such as urea, uric acid, and creatinine in rats fed with 200 mg FB1 revealing apparent kidney toxicity. Hence, our results clearly signify the damaging effects of FB1 on hepatic and renal tissues.

The FB1 toxicity in broilers was further substantiated by the assessment of oxidative stress markers in serum and liver homogenate. The accumulation of free sphingolipid bases due to FB1 toxicity induces an inhibition of complex 1 of the mitochondrial respiratory chain, CYP450, and NADPH oxidase system and therefore stimulates the generation of diverse ROS such as superoxide radical, hydrogen peroxide (Domijan and Abramov, 2011; Mary et al., 2012; Rodrigues and Gomes, 2012). The ingestion of 200 mg FB1 induced a significant increase in ROS and H_2_O_2_ generation, LPO marker (MDA), and carbonyl contents of protein oxidation in serum and liver homogenate in broilers. Recent studies by Theumer et al. (2010) in wistar rats and Kotan et al. (2011) in human lymphocytes indicated that generation of ROS and oxidative stress was a direct consequence of the immunotoxic effects elicited by AFB1 and FB1. Similar results on oxidative stress were observed in rats treated with AFB1 and FB1 (Hassan et al., 2014; Abdellatef and Khalil, 2016) and broiler chicks with FB1 (Poersch et al., 2014). *In vivo* studies of Wistar rats treated with 100 mg FB1, documented a significant increase in MDA level in spleen mononuclear cells, liver and kidney tissues (Theumer et al., 2010; Hassan et al., 2014). Apart from lipids, the other possible major target of oxidative damage is proteins that are further transformed into protein carbonyls. Mary et al. (2012) proposed that 48 h incubation of spleen mononuclear cells with 10 μM FB1 significantly raised the carbonyl content. Also, Domijan et al. (2007) suggested that 200 ng and 50 μg FB1 could cause oxidation of proteins in the kidney of wistar rats.

The histopathological observations of hepatic and renal tissues confirmed the abnormal serum biochemistry, oxidative stress and cellular damage induced by FB1. The intoxicated broilers marked massive destruction of hepatic and renal tissues. Tessari et al. (2006) showed severe damage of liver characterized by disorganization and megalocytosis of hepatocytes, bile duct proliferation, necrosis and inflammation and kidney sections displaying hydropic degeneration in broiler chicks exposed to 50 and 200 mg FB1. Further, Voss et al. (2007) proposed that kidney was the most sensitive organ in Sprague–Dawley and Fischer 344 rats when exposed to FB1, while in BD IX rats, liver was the main target organ.

Our study demonstrates the protective role of a probiotic strain, *L. plantarum* MYS6 (Lp MYS6) against FB1-induced toxicity and tissue damage. The LpMYS6 strain was previously characterized in our laboratory for an array of probiotic attributes, antifungal properties, FB1 detoxification by binding and extraction/purification of low molecular weight antifungal compounds (Deepthi et al., 2016). Probiotic LAB is known to act via diverse mechanisms *in vivo* which include modulation of gastrointestinal physiology by increasing the production of growth factors, competition for nutrients with enteropathogens, bioconversion of available sugars to acids, production of vitamins and organic acids, competitive exclusion for adhesive sites, beneficial immunostimulation of the gut-associated lymphoid tissue, antioxidative and anticancer/antiproliferative activities (Alberto et al., 2007; Oelschlaeger, 2010). Detoxification of mycotoxins by LAB is mainly mediated by the binding of toxin to bacterial cell and is dependent on the cell wall structural integrity of LAB.

In response to probiotic treatment, FB1-challenged broilers showed significant amelioration of toxin ill effects. Broilers of pre-colonization study exhibited a gradual increase in feed intake, body weight and FCR when compared to birds treated with toxin alone. Moreover, the broilers of challenge study showed the highest body weight among all treatment groups. Our results clearly demonstrated that LpMYS6 improved the appetite of FB1-challenged broilers thereby increasing the feed intake and body weight. A recent study by Khalil et al. (2015), reported that *L. delbrueckii* subsp. *lactis* and *Pediococcus acidilactici* significantly helped in improving the body weight and feed intake of rats after three weeks of exposure to 50, 100, and 200 mg concentrations of FB1. Also, our results are in line with the work by Gratz et al. (2006), who demonstrated that *L. rhamnosus* strain GG improved the body weight gain in rats intoxicated with AFB1. LpMYS6 was efficient in reducing the toxicity-induced weight gain in the liver, but there was no significant effect on the relative weight of kidney in all the dietary treatments. With respect to hematological parameters, LpMYS6 efficiently reestablished the RBC count and hemoglobin. The elevated levels of WBC and reduced count of PLT were restored by LpMYS6 administration. Jiang et al. (2014) showed that supplementation of yeast cell wall absorbent had a protective effect on WBC, lymphocytes, PLT, and hemoglobin in broilers fed with mycotoxin-contaminated feed. Further, oral administration of LpMYS6 significantly restored the altered levels of serum parameters such as SGOT, SGPT, creatinine, cholesterol, triglycerides and albumin, both in the pre-colonization and challenge study. The alleviated level of SGPT specifically indicates the rehabilitation of hepatocytes, and also the serum cholesterol. The reduction in elevated levels of cholesterol by LpMYS6 indicate normal lipid metabolism including sphingolipid formation and normalized feed intake. High serum creatinine is an indication of renal trauma. Our strain, LpMYS6 was efficient in reducing creatinine level which may be due to proper catabolism of dietary/tissue proteins and filtration rate in kidney. The above results indicate that LpMYS6 effectively reduced the FB1-induced hepatorenal toxicity. Khalil et al. (2015) reported that co-administration of *L. delbrueckii* subsp. *lactis* and *Pediococcus acidilactici* was efficient in normalizing ALT, AST, albumin, bilirubin levels in rats fed with 200 mg FB1-contaminated diet. Also, Jiang et al. (2014) studied that supplementation of yeast cell wall absorbent to the mycotoxin contaminated diet could significantly improve the serum levels of AST, ALT, ALP, and GGT in broiler chickens.

Furthermore, LpMYS6 effectively scavenged the elevated ROS and H_2_O_2_ in serum and liver homogenate. Also, the probiotic strain remarkably stabilized the altered levels of LPO and PCC in serum and tissue homogenate of liver. Administration of LpMYS6 was observed to significantly reinstate the normal morphology of liver and kidney. Deabes et al. (2012) reported a significant reduction of oxidative status in liver and kidney of aflatoxin-challenged mice treated with *L. rhamnosus* starin GG by increasing the contents of reduced glutathione (GSH) and superoxide dismutase (SOD). Further, a study by Abdellatef and Khalil (2016), demonstrated the ameliorative effects of *L. delbureckii* subsp. *lactis* DSM 20076 and *Pediococcus acidilactici* NNRL B-5627 on fumonisin B1-induced hepatoxicity and nephrotoxicity in rats.

LpMYS6 induces its protective effects probably by binding FB1 in the chicken crop or gastrointestinal tract and consequently reducing the bioavailability of FB1. This is supported by our previous observation on the *in vitro* binding ability of *L. plantarum* MYS6 to FB1 toxin wherein the per cent removal of FB1 was 32.9% and 61.7% in 2 and 4 h of incubation time, respectively (Deepthi et al., 2016). Several studies suggest that binding is the main mechanism of detoxification of mycotoxins by LAB (Gratz et al., 2007; Hathout et al., 2011; Abbes et al., 2016; Abdellatef and Khalil, 2016). However, the binding mechanism itself is not thoroughly understood. Zoghi et al. (2014) reported that probiotic LAB binds to the toxin due to the adhesive nature of S-layer proteins in their cell wall. Shetty and Jespersen (2006) suggested that carbohydrate-rich mannoproteins or glucans of LAB are involved in their binding to mycotoxins. Recently, Huang et al. (2017) suggested that *L. plantarum* C88 could effectively detoxify aflatoxin by suppressing the expression of cytochrome P450 1A2 and CYP 3A4 to decrease the production of AFBO (exo-AFB1-8,9-epoxide) and activate GST A3 through Nrf2 signaling pathways. This in turn improves GSH-conjugating activity and reduces toxin mediated oxidative stress. The majority of studies on detoxification of mycotoxins by LAB are aflatoxin-oriented with very limited data existing for LAB-mediated amelioration of FB1-induced toxicity and oxidative stress in broilers. In this context, we made an attempt to evaluate the protective effects of a potent probiotic strain, *L. plantarum* MYS6 against FB1 toxicity and oxidative stress in broilers. Our results showed that protective effects of LpMYS6 were significantly high in challenge study when compared to pre-colonization study. This indicates that the binding of FB1 occurs immediately after administration of LpMYS6 and pre-colonization of LpMYS6 might delay or decrease the binding process. Our outcome is in line with a recent finding by Huang et al. (2017) who reported that *L. plantarum* C88 effectively binds to AFB1 within 2 h post dose in mice and excreted in high levels as fecal AFB1 and lactobacilli. Nevertheless, systemic studies are still needed to understand the precise binding mechanism of FB1 to LAB.

Toxin binders are inert indigestible adsorbents widely employed in the poultry feed industry as detoxicification method. Several studies are available suggesting the application of TOXBs such as HSCAS, clay products, bentonites, zeolites, activated carbon etc (Huwig et al., 2001; Galvano et al., 2001; Avantaggiato et al., 2004; Kabak et al., 2006; Jouany, 2007; Phillips et al., 2008; Devreese et al., 2013; Agboola et al., 2015) as an effective method to minimize mycotoxin-induced toxicity. Interestingly, we found that TOXB was less effective in sequestering the toxin. Moreover, they seemed to induce oxidative stress and organ damage to some extent in our study.

In summary, the present investigation clearly demonstrates the FB1-induced toxicity, oxidative stress and vital organ damage in broilers. Oral administration of *L. plantarum* MYS6 to broilers significantly mitigated the FB1-induced toxicity and organ damage. *L. plantarum* MYS6 also significantly reinstated the imbalanced serum biochemical parameters, oxidative markers and hepatic and renal tissue damage. *L. plantarum* MYS6 was more effective in sequestering the toxin when compared to the commercially available TOXB and had protective health benefits. The protective role of *L. plantarum* MYS6 may be due to its FB1-binding capacity thereby reducing the toxin bioavailability in broilers. To the best of our knowledge, the present study is the first of its kind in employing a probiotic lactic acid bacterium to reduce the effects of FB1-induced oxidative stress and organ damage in broilers. The future prospect of the study is to understand the changes in gut microbiota, mechanism of interaction between LAB and FB1 at molecular level and developing a functional probiotic feed for broilers.

## Author Contributions

MYS, KSG, and BVD conceived and designed the research; BVD, RS, KPR, and ND performed the experiments and animal work; NKD supervised animal work and necropsy; BVD, RS, NKD, KSG, and MYS analyzed the data; BVD wrote the paper and MYS edited the paper.

## Conflict of Interest Statement

The authors declare that the research was conducted in the absence of any commercial or financial relationships that could be construed as a potential conflict of interest.
